# Influence of shear force on ex vivo expansion of hematopoietic model cells in a stirred tank bioreactor

**DOI:** 10.1186/s13036-023-00358-4

**Published:** 2023-06-05

**Authors:** Sofia Mohseni, Ali Baradar Khoshfetrat, Reza Rahbarghazi, Shahla Khodabakhshaghdam, Leila Shafiei Kaleybar

**Affiliations:** 1 Chemical Engineering Faculty, Sahand University of Technology, Tabriz, 51335-1996 Iran; 2 Stem Cell and Tissue Engineering Research Laboratory, Sahand University of Technology, Tabriz, 51335-1996 Iran; 3 Stem Cell Research Center, Tabriz University of Medical Sciences, Tabriz, Iran; 4 Department of Applied Cell Sciences, Faculty of Advanced Medical Sciences, Tabriz University of Medical Sciences, Tabriz, Iran; 5 Department of Chemical Engineering, University of Bonab, Bonab, Iran

**Keywords:** Agitation rate, Human pro-monocytic U937 cells, Ex vivo cell proliferation, Stirred bioreactor, Shear rate

## Abstract

To evaluate shear stress influence on ex vivo expansion of hematopoietic cell lineages for clinical application, in this study, human pro-monocytic cell (namely U937 cell line) was selected as a hematopoietic stem cell (HSC) model and cultured in suspension mode at two different agitation rates (50, 100 rpm) in the stirred bioreactor. At the agitation rate of 50 rpm, the cells achieved higher expansion folds (27.4 fold) with minimal morphological changes as well as apoptotic cell death, while at 100 rpm the expansion fold decreased after 5-day of culture in suspension culture in comparison with static culture and reached 24.5 fold at the end of the culture. The results of glucose consumption and lactate production were also in agreement with the data of fold expansion and indicated the preference of culture in the stirred bioreactor when agitated at 50 rpm. This study indicated the stirred bioreactor system with an agitation rate of 50 rpm and surface aeration may be used as a potential dynamic culture system for clinical applications of hematopoietic cell lineage. The current experiments shed data related to the effect of shear stress on human U937 cells, as a hematopoietic cell model, to set a protocol for expansion of HSCs for biomedical applications.

## Introduction

Hematopoietic stem cells (HSCs) and progenitors are considered suitable cell sources for the treatment of different diseases such as blood and immune system deficiencies [1, 2]. However, the numbers of available cells are insufficient to help all patients benefit from treatment with HSCs, and progenitors [1, 3]. Successful ex vivo expansion of HSCs and progenitors is of particular importance in the development of therapies for patients with hematological disorders. It was suggested that HSCs expansion on 2D static culture systems leads to the formation of gradients of nutrients, temperature, pH, and dissolved oxygen that can affect cell features over time during culture [4, 5]. Numerous strategies have been assigned to the ex vivo expansion of HSCs to improve engraftment time and reduce the graft failure rate [6].

Bioreactors develop a dynamic cultivation system within a controlled environment providing the physicochemical requirements for the expansion of stem cells [7]. So far, different types of bioreactors were developed and investigated for HSC expansion. Bioreactors such as stirred tanks, fixed beds, airlifts, perfusion chambers, rotating wall vessels, and hollow fiber have been recently studied for HSC cultivation [3, 8]. Among various types of bioreactors that are commercially available, stirred tank bioreactor (STB) has attracted much interest in stem cell proliferation due to its unique characteristics such as relatively simple and easy to operate, different modes of operation, and better mass transfer capability [9–11].

On the other hand, there is a close relationship between the bioactivity of hematopoietic cells with shear stress, making aeration and agitation rates critical parameters in the STB systems to maintain HSCs functionalities [12]. To decrease the shear stress in the culture of hematopoietic cells, we have already used the proliferation chamber connected to the STB system as a conditioning vessel [3, 13]. Recently, we have also designed a conceptual bioprocess based on the stirred tank bioreactor with a sequencing batch aeration system and evaluated the bioreactor performance for one-step clinical mass production of hematopoietic cells [7, 14]. Although the results of the STB systems for hematopoietic cell expansion are hopeful, detailed cell proliferation and function studies are still needed in the bioreactor system to offer suitable strategies to expand hematopoietic stem cells *in vitro* for clinical applications.

Possible impacts of sheer stress on non-adherent stem cells behavior such as hematopoietic cells inside stirred tank bioreactors have not been examined in detail. Indeed, it is important to understand how to optimize bioprocessing parameters to maximize cell expansion without quality loss and therapeutic potency. Reports show agitation speed plays a critical role in fluid shear in stirred bioreactors [15]. To investigate the influence of shear forces on hematopoietic cell self-renewal expansion, in this work, the hematopoietic model cells (U937 cells) were cultured in stirred bioreactor at two different agitation rates of 50 and 100 rpm. Mechanical characteristic quantities and their impacts on the proliferation behavior of hematopoietic model cells were also evaluated at different agitation rates. Data from the current study can help us to assess the possible effect of shear stress on the dynamic activity of hematopoietic cell lineages using several biochemical parameters before application for stem cell-based therapies.

## Materials and methods

### Materials

Human pro-monocytic cell line (U937) was purchased from (Institute Pasture, Iran). Sodium alginate (medium viscosity, viscosity > 2000 cps), gelatin, calcium chloride, citrate sodium, and HEPES were prepared from Sigma-Aldrich. Roswell park memorial institute medium (RPMI 1640, Invitrogen), penicillin/streptomycin (pen/strep), fetal bovine serum (FBS), phosphate-buffered saline (PBS), and 0.25% Trypsin-EDTA solution were obtained from Gibco (France).

### Bioreactor system

**Fig. 1:**
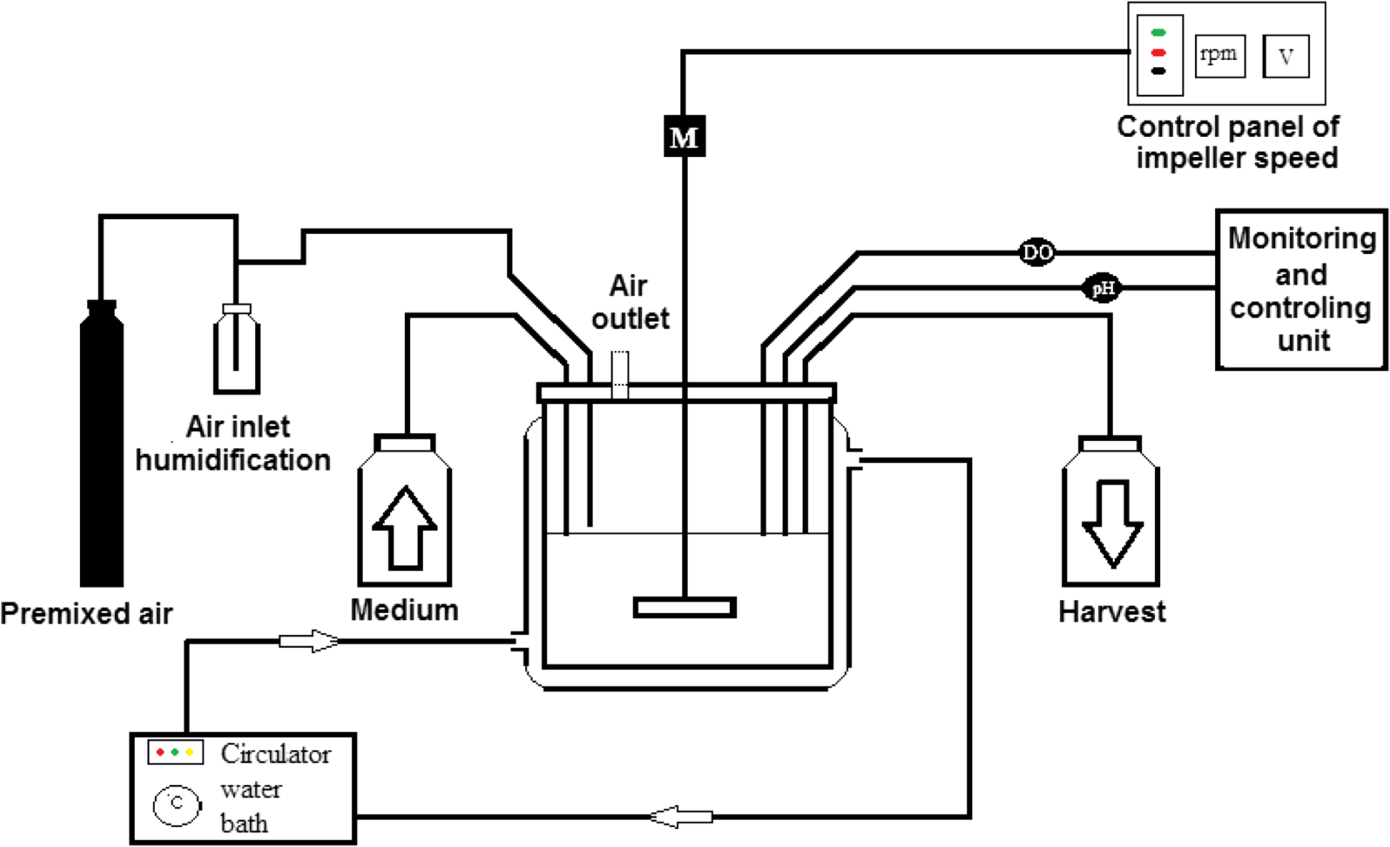
Schematic of the stirred bioreactor developed

Dynamic cultures were performed in a lab-scale stirred tank bioreactor. The cylindrical glass bioreactor with 100 ml working volume was equipped with a flat blade paddle impeller rotated by an external motor (Fig. 1). Temperature was under control at 37°C by using a water circulator having temperature controller (UC5000, Sahand Azar Co.). The culture medium was aerated by the surface aeration method. The inlet gas (5% CO_2_ and 95% air) entered the bioreactor at an aeration rate of 0.1 vvm after passing through a humidifier. The aspect ratio, the medium height (H) to the diameter of the bioreactor (D), was 0.46. The diameter of impeller (d) was 3.25 cm, resulting in the ratio of the diameter of the stirrer’s blade to the diameter of the vessel (d/D) of 0.5.

### Cell culture

Human pro-monocytic U937 cells were used as the hematopoietic cell model to inoculate static and dynamic culture systems. RPMI1640 supplemented with 10% (v/v) FBS and 100 U/ml antibiotics were used as a culture medium. Experiments were carried out at an initial seeding density of 10^5^ cells/ml in both the bioreactor and T- flask for 7 days. The T-flasks were incubated at 37°C without agitation in a humidified 5% CO_2_ incubator. The 50% of the culture medium was replaced with fresh medium on days 1, 3, and 5, and the used medium was stored at -20°C for analysis. At determined culture days, the concentrations of glucose and lactate, and the pH levels of the medium were measured. The agitation rates of 50 and 100 rpm were studied for the suspended cells.

### Shear stress consideration

Agitation is essential for suspending cells and obtaining the homogeneity of nutrition and oxygen in stirred bioreactors. It has been stated that excessive agitation is a cause of death and dedifferentiation of cells [16]. To investigate the shear stress, the maximum threshold, which depends on the cell type, should be checked because of its harmful effects on the cells at higher values. Based on the previous studies, the maximum shear stress imposed on a cell/aggregate could be estimated by the following equation [16–19]:

1 $${{\tau }_{max}=5.33\rho \left(\varepsilon \vartheta \right)}^\frac{1}{2}$$

where *ρ* is the medium density (kg/m^3^), *υ* is the kinematic viscosity of the medium (m^2^/s) and *ε* is the power dissipated per unit mass (W/kg) which is calculated by Eq. 2:

2 $$\varepsilon =\frac{P}{{V}_{\mathrm{L}}.\rho }$$

where *P* is the power consumed (W) and *V*_L_ is the bioreactor working volume (m^3^). The consumed power can be obtained from Eq. 3 [20]:

3 $$P={N}_{p}{N}^{3}{d}^{5}\rho$$

where *N* is the impeller speed (rps) and *d* is the impeller diameter (m). The value of the dimensionless power number (*N*_*P*_) which is a weak function of impeller geometry and Reynolds number (*Re* > 1000), can be calculated from the Nagata correlation [20].

The physical properties of water can be considered for the culture medium due to the similarity of fluid properties of the culture medium to those of water.

The damages caused by shear stress make it necessary to select the appropriate mixing rate. Additionally, shear stress depends on the geometric characteristics of the vessel as well as the impeller and the physical properties of the culture medium. Integrated shear factor (ISF) defined by Sinskey et al. [21] for mammalian cells was used to examine the damage caused by the shear between the impeller and the walls of the vessel (Eq. 4) [19, 22]:

4 $$ISF=\frac{2\pi Nd}{D-d}$$

where *D* and *d* are the diameters of the impeller and the vessel, respectively.

### Metabolic rates

The bioreactor was operated in the batch mode, so the following is the cell’s mass balance for the whole process [17]:

5 $$M.B. :In-Out+Conv. =ACC$$

6 $$M.B. \left(X\right):0-0+\left(\mu .{C}_{x}\right).V=d({C}_{x}.V)/\mathrm{dt}$$

Assuming the logarithmic growth for the cell cultures and constant working volume (*V*) (Eq. (6)), the cell density (*C*_x_) could be obtained from Eq. (7).

7 $${C}_{x}={C}_{x0}\mathrm{exp}(\mu t)$$

Where* C*_x0_ is the initial cell density (cells/ml) and *μ* is the specific cellular growth rate (h^-1^) which is obtained from the cellular growth profile, and *t* is time (h).

Considering glucose as the main source of energy production and lactate as the major product of cells, the mass balance for each batch of the medium could be written as Eq. (8) [17]:

8 $$M.B.\left(S\right):0-0+\left({\pm q}_{met }.{C}_{x}\right).V=d({C}_{met}.V)/dt$$

The specific metabolite rate (*q*_met_) for glucose consumption and lactate production in the batch process was assumed to equal an average constant value measured for each cell type. Therefore, at constant volume, the mass balance could be written as [22, 23]:

9 $${q}_{met} {C}_{x}=\frac{{dC}_{met}}{dt}$$

Which, *t* is the period, and *C*_x_ is the average viable cell density during the same period (cells/ml).

### Quantitative analysis of cell morphology

The cell morphology was quantitatively evaluated by a parameter named cell roundness, Rc as described elsewhere [13, 24]. Briefly, the images of cells were captured by using a light microscope (Model: BX51; Olympus, Japan). The roundness of cells was evaluated with image analyzing software (Image J, version 1.52). In this study, the cells which have *R*_c_ > 0.8 were considered round. The frequency of round-shaped cells (*f*_r_) was determined as follows [25, 26]:

10 $$f\mathrm r=\frac{\mathrm{number}\;\mathrm{of}\;\mathrm{cells}\;\mathrm{having}\;R_c>0.8}{\mathrm{total}\;\mathrm{number}\;\mathrm{of}\;\mathrm{cells}\;\mathrm{examined}}$$

### Flow cytometry analysis and cell counting

The total number of cells was determined by staining with trypan blue with a hemocytometer at days 0, 3, 5, and 7. The count of suspension cells was performed under an optical microscope (Olympus CKX31). To elucidate the possible effect of the dynamic culture method on the induction of apoptotic cell death, flow cytometry analysis was performed.

### Measuring cell apoptosis using flow cytometry analysis

To test whether the exposure of U937 cells to shear force can promote apoptotic change, we performed a flow cytometric analysis of Annexin-V. To this end, cells were collected after 7 days and washed twice with PBS, and fixed with 4% paraformaldehyde solution. After that, cells were permeabilized using 0.05% Triton X100 and blocked with 1% BSA solution. Using FITC labeled anti-human Annexin-V antibody (Sigma-Aldrich), the cells were stained and washed twice with PBS. Cells were analyzed using the FACSCalibur system (BD Biosciences) and FlowJo software (version 7.6).

### Statistical analysis

Experiment results were presented as mean ± SD. Difference among samples were considered statistically significant by the confidence interval of 95% (*p* ˂ 0.05).

## Results

### Influence of shear force on hematopoietic cell model proliferation and culture in STB

**Fig. 2:**
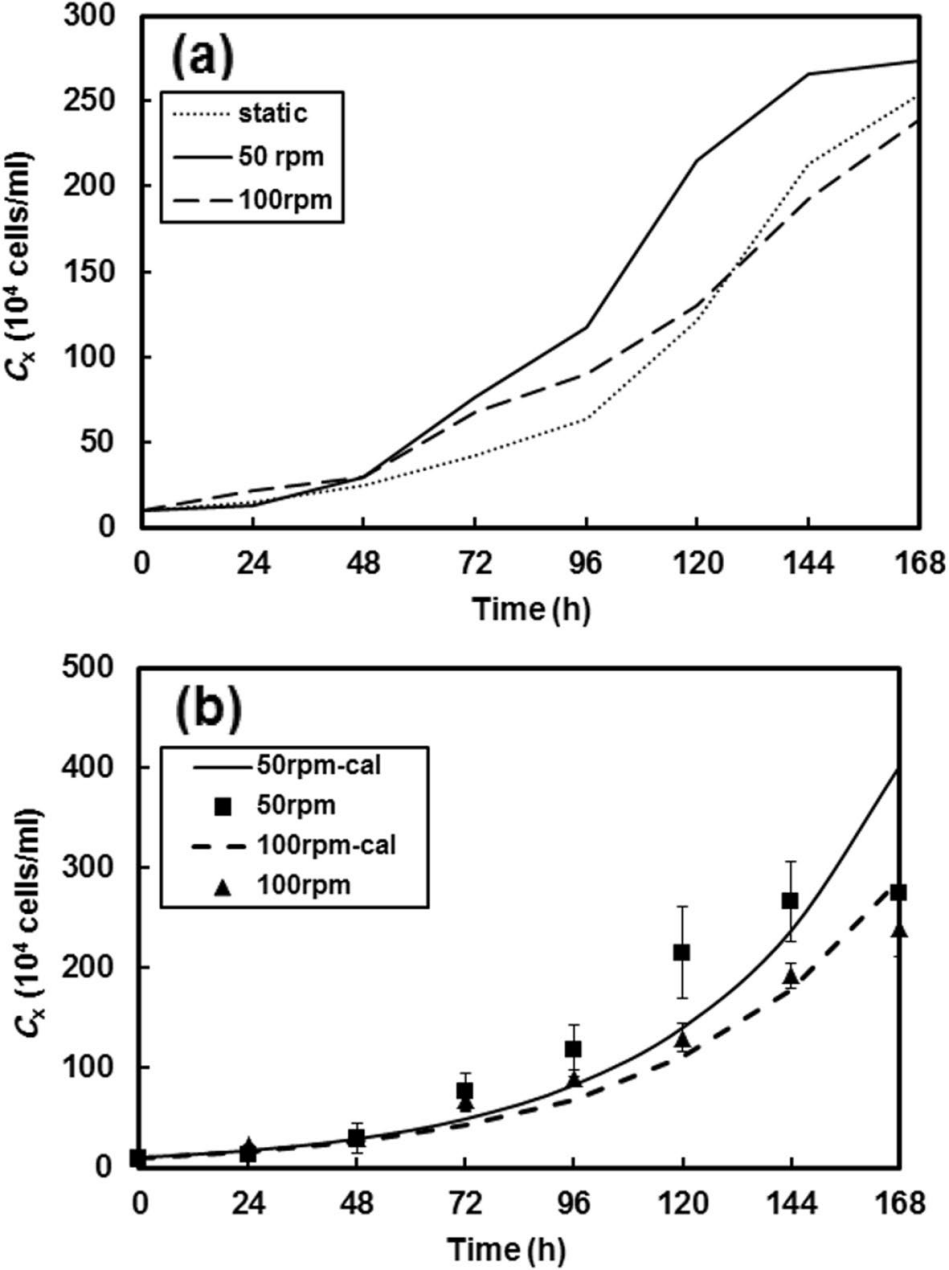
Growth profiles of U937 cells in different culture modes (**a**), and exponential cell growth profile compared with experimental data at 50 rpm and 100 rpm (**b**)

The model cells were cultivated in the stirred bioreactor with an agitation rate of 50 and 100 rpm, in suspension culture mode. Changing agitation rate affected considerably the proliferation behaviors of the hematopoietic cultured in the stirred bioreactor system, as shown in Fig. 2a. According to the results of MTT assays, the metabolic activity of the cells cultured at 50 rpm demonstrated a higher expansion fold than the static culture during the cultivation period. The cells showed a specific growth rate of 0.028 h^-1^ and were expanded up to 27.4-fold as the final cell density reached 2.74×10^6^ cells/ml at the end of the culture period. Total cell expansion at 100 rpm was significantly less than that of the static culture from day 6, as the cells showed the specific rate of 0.022 h^-1^ and were expanded up to 24.5-fold, reaching 2.45×10^6^ cells/ml at the end of the culture. As illustrated in Fig. 2b, during the culture period, the experimental results confirmed the calculated logarithmic growth.

**Fig. 3:**
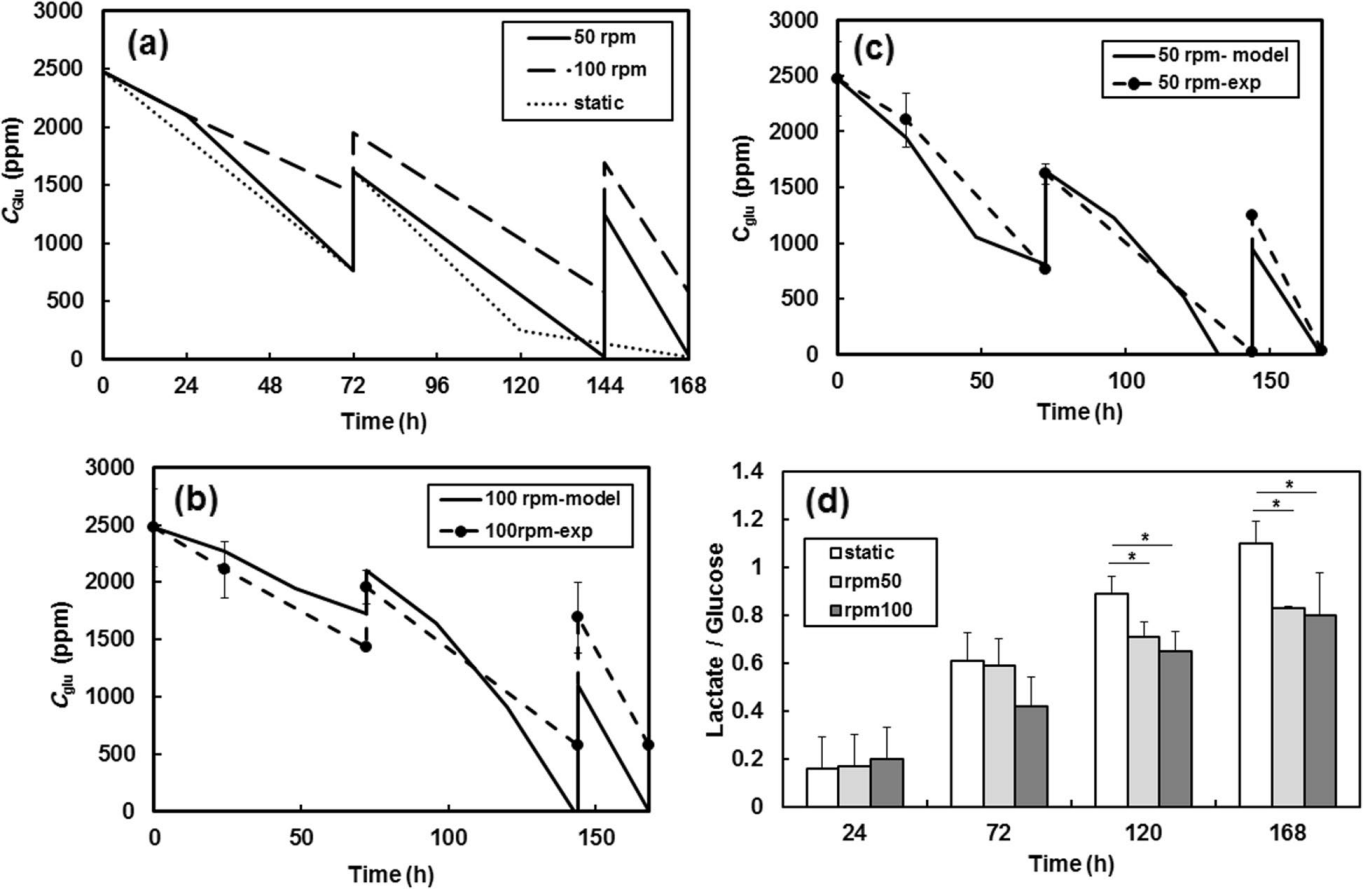
Glucose concentration at the determined days during culture days (**a**); comparison of model prediction with the experimental data at 50 rpm(**b**) and 100 rpm (**c**), and the ratio of lactate production to glucose consumption at determined days (**d**) (* *p* < 0.05)

Glucose and lactate concentrations were also measured as two important metabolites within the culture system during the culture days. As shown in Fig. 3a, the glucose concentration of the culture medium at static culture and 50 rpm agitation rate decreased considerably during the culture days, indicating the proper conditions for cell growth. The glucose concentration profile at 100 rpm revealed higher values compared to static culture and 50 rpm agitation rate. The metabolite alteration results were consistent with the viability of the cells. Interestingly, the glucose consumption at different agitation rates was well-predicated (0.92 ≤ *R*^*2*^ ≤ 0.98) with the obtained model (Eq. 8), as shown in Fig. 3b,c.

The ratio of lactate production to glucose consumption was also used as another index for cellular metabolism measurement (Fig. 3d). It was observed that during the beginning of the experiment, the ratios were similar for all systems. In Fig. 3d, dynamic culture systems exhibited significantly smaller overall ratios compared to the static culture, indicating better oxygenation than static culture [27, 28]. It is generally reasonable to conclude that cells cultured at 50 rpm with lower shear stress had more variation in nutrient profiles and metabolites. In addition, no significant alteration in pH value was observed during the 7-day culture period in dynamic culture (data not shown).

### Influence of shear force on hematopoietic cell model morphology in STB

**Fig. 4:**
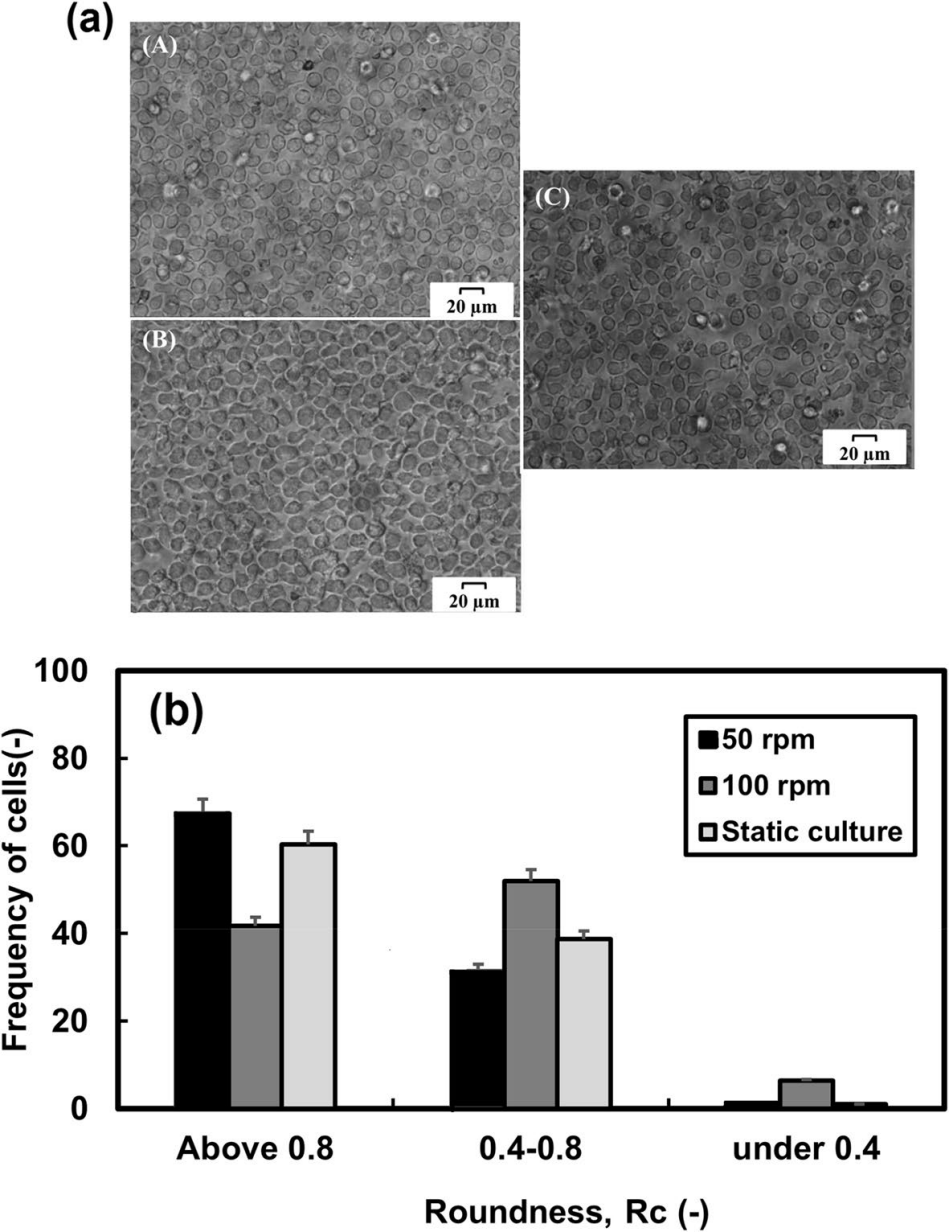
Phase-contrast microscopy images of cells at 50 rpm (A), 100 rpm (B), T-flask (C), at 7- day (a) and distribution of cell roundness (*R*c) at different cell culture systems. The results were obtained from 100 cells at the end of culture day (b)

Cells morphology analysis was evaluated to study the effect of shear stress on the appearance of cells. As shown in Fig. 4a, the cells cultured at 50 rpm agitation rate and T-flask had a round-shape appearance, while the cells cultured at 100 rpm were deformed and appeared to be stretch-shaped. The threshold value for *R*_c_ was set at 0.8 to distinguish round-shaped cells from stretch-shaped ones. The frequencies of the round-shaped cells at 50 rpm, 100 rpm, and T-flask approximately were attained at 67%, 41%, and 60%, respectively (Fig. 4b). The results revealed that the cell morphology changed more to stretch shape with increasing agitation rate in the bioreactors.

### Influence of shear force on apoptotic hematopoietic cell model death in STB

**Fig. 5:**
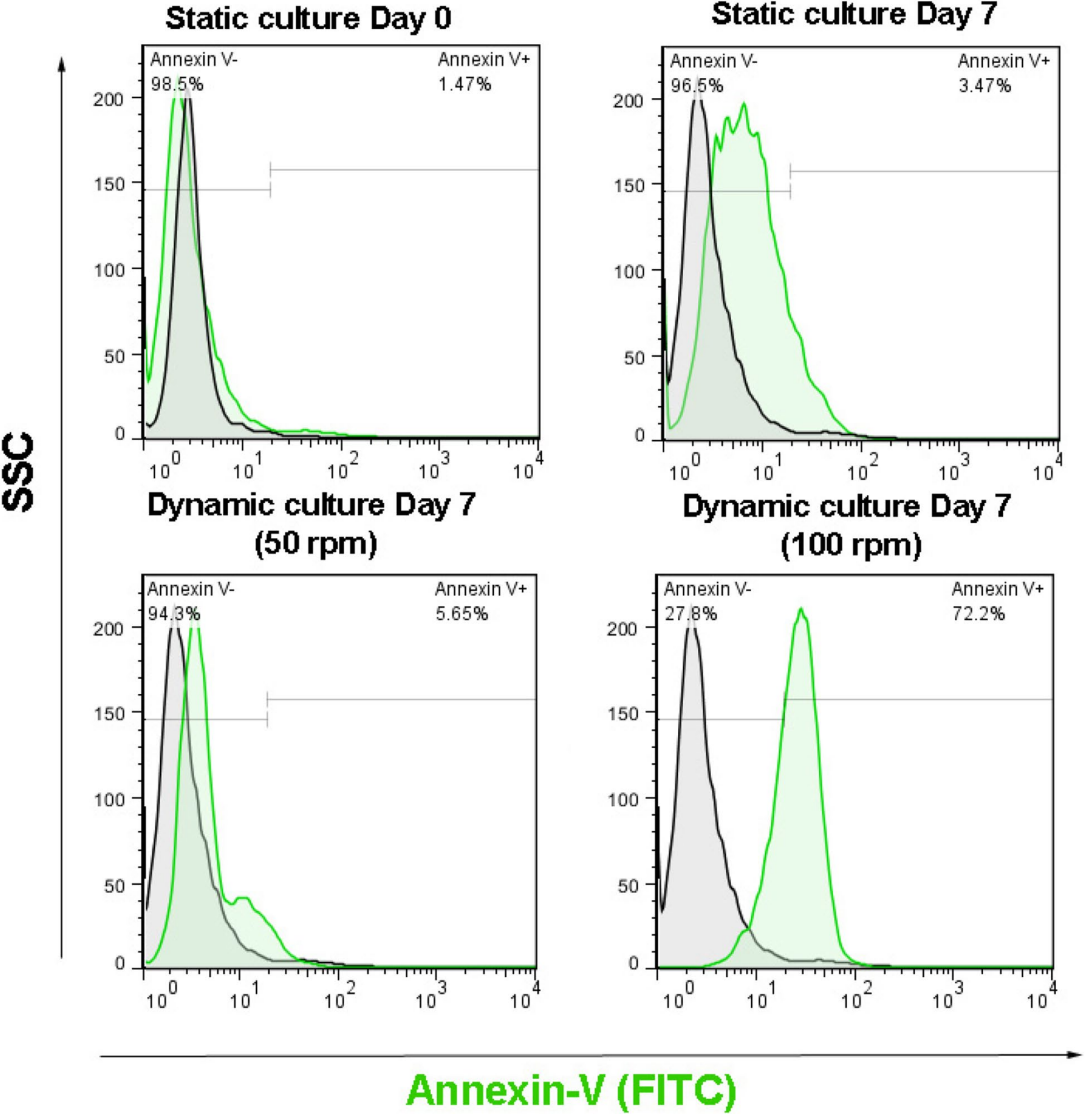
Flow cytometry analysis results

To elucidate the possible effect of the dynamic culture method on the induction of apoptotic cell death, flow cytometry analysis was performed. It was found that over 97±1.3% of U937 cells exhibited no signs of apoptosis in the static culture system. The percentage of apoptotic cells increased from 1.47±0.05% on day 0 to 3.47±0.32% on day 7. In the dynamic culture at 50 rpm, it was found a slight increase in the percentage of apoptotic cells, which did not reach statistically significant levels. However, the introduction of 100 rpm in the dynamic culture system promoted apoptotic changes in U937 cells, about 72±6.48%, compared to the time-matched control and cells from the 50 rpm group on day 7. The data showed a distinct effect of a dynamic culture system with a specific rpm on the survival rate of U937 cells in-vitro (Fig. 5).

## Discussion

Agitation is required to keep the cells from sedimentation and to assure a homogeneous environment for cell growth in dynamic cultures [29]. The mixing within stirred bioreactors can generate high shear stresses depending on the rotation speed and vessel design [30]. The impact of stress generated by agitation on cell culture is usually analyzed by Kolmogorov’s theory of isotropic turbulence [8, 9, 30–32]. It is clear from the literature that damage to cells increases with increasing aggregation size, agitation rate, and amount of cell loading. Indeed, the damage becomes significant when the eddy size is about two-thirds of the size of the aggregations, or smaller [32–34]. The primary cellular damage appears to result from direct interaction between cell or cell aggregates and turbulent eddies, collisions between cells and collisions between impeller and cells.

In this study, we examined the effect of two different shear stress values on model HSC proliferation behaviors. The calculations for hydrodynamic parameters of the stirred bioreactor system are shown in Table 1. The estimated eddy size (*λ*_K_), at 50 and 100 rpm were calculated at 280 and 166 µm, respectively. In this study, since the size of cells is very smaller than the size of the smallest eddies in suspension culture, the interaction between cells and eddies is not significant and cells just follow the local flow of the culture medium. It seems that in the suspension culture at 100 rpm, an increase in wall shear stress at such a high agitation rate and collisions of cells against the impeller or the bioreactor internals result in damage to cells so that the cell expansion decreased after 5 days compared to the static culture [23].

**Table 1:** The values of hydrodynamic parameters at two different rates

**Impeller speed, N (rpm)**	**50**	**100**
Reynolds number, Re	867.69	1763.93
Power number, *N*_p_	0.78	0.69
Energy dissipated per unit mass, *ε* (m^2^s^-3^)	1.62×10^-4^	1.32×10^-3^
Maximum shear stress, *τ*_max_ (pa)	0.068	0.193
Integrated shear factor, ISF (s^-1^)	5.46	10.49
Size of smallest eddy, *L* (µm)	280	166
Volumetric oxygen mass transfer coefficient, *K*_l_*a* (h^-1^)	0.89	3.63

Studies indicate that shear stress affects the phenotype of attached as well as suspendable cells. Endothelial cells become elongated and alter their morphology, function, and gene expression when they exposure to shear stress [35, 36]. We have already shown that the *R*_c_ parameter may be used to monitor indirectly the stress level exerted on the cells in the dynamic culture systems [13]. The quantitative morphological analysis of the cells in the present study also revealed that the cell morphology altered more to stretch shape (lower *R*_c_ values) under the dynamic cultures, particularly at the agitation rate of 100 rpm. The morphology alteration of the cells in the stirred bioreactor, therefore, can be considered a high-shear stress influence on the cells.

The shear stress values exerted on the cells at the agitation rates of 50 and 100 rpm in the bioreactor system were calculated at 0.068 and 0.192 Pa, respectively. Interestingly, the threshold shear stress for HSCs proliferation and function has been reported 0.092 Pa [23]. The amount of shear stress observed at 100 rpm, therefore, is higher than the threshold shear stress for HSCs, resulting in more changes in cell morphology and function.

In the study of agitation speed influence on the expansion of HSCs cells, Jing et al. [11] concluded that HSCs must be cultured at a relatively low agitation speed (30 rpm) because high agitation speed (80 rpm) caused significant cell loss and rapid cell differentiation. In addition, they stated that a culture period of about 6-9 days showed better conditions for getting higher expansion folds and maintaining hematopoietic functionality. Hosseinizand et al. [23] have also studied the effects of agitation on umbilical cord blood hematopoietic stem cell growth and differentiation. Various agitation rates (20, 40, and 60 rpm) were compared in spinner-flask and static culture. Among the culture systems, the spinner flask with a 40 rpm agitation rate showed good cell expansion with about a 5-fold increase in total cell number.

Consistent with the studies, the model HSCs cultured in the stirred bioreactor at 50 rpm in the present study revealed a higher proliferation rate with minimal morphological changes as well as apoptotic cell death. The findings of the current study demonstrate that the stirred bioreactor system with an agitation rate of 50 rpm and surface aeration may be considered a potential dynamic culture system for clinically-required HSCs, although further experiments are needed in this regard.

## Conclusion

In the present study, the proliferation of U937 as a model of hematopoietic stem cells was studied at different agitation conditions in the stirred bioreactor. This study showed that the use of dynamic culture was able to increase cell expansion compared to static culture. However, various types of fluid shear stress generated in the stirred bioreactor will influence the expansion and differentiation potential of hematopoietic stem cells. Therefore, the optimal agitation would favor regulating the culture environment to expand cells effectively. Here, we used human pro-monocytic cell line U937 as a hematopoietic cell model for expansion inside the developed stirred bioreactor. How the metabolic signature of these cells can be applied to normal HSCs and progenitors should be addressed. In conclusion, further studies are needed for defining the optimum mechanical forces for hematopoietic stem cell culture in the stirred bioreactor. Our future work may include optimization studies of the bioreactor system as well as microencapsulated cell culture at various agitation rates to determine the best culture condition.

## Data Availability

All data generated or analyzed during this study are included in this published article.
